# Functional characterization of all‐trans retinoic acid‐induced differentiation factor (ATRAID)

**DOI:** 10.1002/2211-5463.13685

**Published:** 2023-09-07

**Authors:** Roya Mehrasa, Ileana Cristea, Cecilie Bredrup, Eyvind Rødahl, Ove Bruland

**Affiliations:** ^1^ Department of Clinical Medicine University of Bergen Norway; ^2^ Department of Medical Genetics Haukeland University Hospital Bergen Norway; ^3^ Department of Ophthalmology Haukeland University Hospital Bergen Norway

**Keywords:** ATRAID, endosomes, Golgi, isoform, N‐glycosylation, RAB11

## Abstract

All‐trans retinoic acid‐induced differentiation (ATRAID) factor was first identified in HL60 cells. Several mRNA isoforms exist, but the respective proteins have not been fully characterized. In transfected cells expressing Myc‐Flag‐tagged ATRAID Isoform (Iso) A, B, and C, Iso C was found to be expressed at high levels, Iso A was found to be expressed at low levels due to rapid degradation, and the predicted protein expressed from Iso B was not detected. Iso C was present mainly in an N‐glycosylated form. In subcellular fractionation experiments, Iso C localized to the membranous and nuclear fractions, while immunofluorescence analysis revealed that Iso C is located close to the plasma membrane, mainly in cytoplasmic vesicles and in the Golgi area. We confirm that Iso C colocalizes to some extent with endosomal/lysosomal markers LAMP1 and LAMP2. Furthermore, we show that ATRAID co‐localizes with RAB11, a GTPase associated with recycling endosomes and implicated in regulating vesicular trafficking.

## Graphical abstract

ATRAID isoform A and C are indicated. Iso C is N‐glycosylated and found in high densities close to the nucleus where it colocalizes with Golgi markers GALNT2 and GM130. Iso C colocalizes with proteins LAMP1, LAMP2, GALNT2, and GM130 as well as RAB11, a marker for recycling endosomes. Iso A is rapidly degraded by a proteasomal degradation pathway.

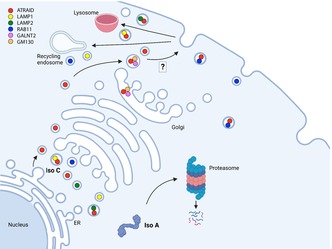

**Abbreviations**
Aa: amino acid
ARPE‐19: arising retinal pigment epithelial cells 19
ATRAID: all‐trans retinoic acid‐induced differentiation factor
GALNT2: polypeptide N‐acetylgalactosaminyltransferase 2
HEK 293: human embryonic kidney cells 293
Hum‐Fib: human immortalized fibroblasts
Iso A: isomer A
Iso B: isomer B
Iso C: isomer C
KEAP1: Kelch‐like ECH‐associated protein 1
LAMP1: Lysosomal‐associated membrane protein 1
LAMP2: Lysosomal‐associated membrane protein 2
NBPs: nitrogen‐containing‐bisphosphonates
NELL1: Neural EGFL like 1
NRF2: Nuclear factor erythroid 2–related factor 2
RAB11: Ras‐associated binding protein 11
RPE1: Retinal pigment epithelial cells 1
SLC37A3: Solute Carrier family 37‐member A3
TOMM20: Translocase of Outer Mitochondrial Membrane 20

All‐trans retinoic acid‐induced differentiation factor (ATRAID) factor, also known as APR3, received its name as its mRNA expression was firmly increased in HL‐60 cells treated with all‐trans retinoic acid (ATRA) [1]. All‐trans retinoic acid‐induced differentiation factor is located at chromosome 2p23.3 in humans, while its mouse ortholog (84% shared homology) is located at chromosome 5 [2]. According to NCBI, only two transcripts of this gene, NM_001170795 (Isoform C [Iso C]) and NM_016085 (Isoform A [Iso A]) are translated (Fig. S1). The most striking difference between these isomers is a postulated N‐terminal signal peptide in Iso C that is lacking in Iso A. Isoform B (Iso B), NM_080592, has been retracted from NCBI due to lack of evidence of a translated protein product.

According to InterPro [3] and PROSITE [4], ATRAID contains several predicted functional domains: a signal sequence at the N terminus, a Toll‐like leucine‐rich repeat followed by an EGF‐domain, a transmembrane region in the C‐terminal part, and finally a short C‐terminal end (Fig. S2). In an in‐silico analysis, Ding and coworkers found that ATRAID contains a potential signal peptide in the first 25 N‐terminal amino acids (aa) with a potential cleavage site at position 25–26 [2]. In addition, they observed three putative N‐glycosylation sites at position 39 73 133 and one putative transmembrane domain (residues 192‐214). Furthermore, *in silico* predictions have suggested that ATRAID is a lipophilic and highly hydrophobic protein [2]. Taken together, it is believed that ATRAID is a glycosylated transmembrane protein, but which membrane system ATRAID is localized to, and its role, remain to be settled.

All‐trans retinoic acid‐induced differentiation factor has been associated with several cellular processes. Overexpression of ATRAID is reported to inhibit the expression of cyclin D1 arresting cells in G1/S phase [5]. In osteoblasts, this effect of ATRAID likely occurs through binding of ATRAID to neural EGFL‐like 1 (NELL‐1), leading to differentiation accompanied by decreased proliferation of the cells [6]. All‐trans retinoic acid‐induced differentiation factor has been associated with the release of nitrogen‐containing bisphosphonates (NBPs) from the lysosomal compartment to the cytosol, by forming a complex in the lysosome with Solute Carrier family 37 member A3 (SLC37A3) [7] and shown to be required for the protective effect of NBPs in bone diseases [8]. Also, ATRAID has been reported as a lysosomal integral membrane protein by Bagshaw and coworkers [9].

All‐trans retinoic acid‐induced differentiation factor has been observed in mitochondria in transfected arising retinal pigment epithelial cells 19 (ARPE‐19) cells, involved in regulating mitochondrial phase II enzymes and the cellular redox status by binding to the complex of nuclear factor erythroid 2–related factor 2 (NRF2) and Kelch‐like ECH‐associated protein 1 (KEAP1) [10]. Han and coworkers reported increased the expression of ATRAID in RPE cells from aged mice. In ARPE‐19 cells undergoing chronic oxidative stress, both increased levels of ATRAID and premature senescence were observed [11]. Interestingly, ATRAID was reported as one of several membrane‐associated proteins found to interact with the HIV‐1 Nef protein in a split‐ubiquitin based yeast two‐hybrid screen targeting integral membrane proteins [12].

In this study, we have examined ATRAID protein expression in transduced and nontransduced cell lines. We find that the main expressed isoform of ATRAID is Iso C. Iso A is expressed but rapidly degraded by the proteasomal degradation pathway. The postulated Iso B protein was not detected. Moreover, ATRAID Iso C was found to be heavily N‐glycosylated, and N‐deglycosylation was required to detect endogenously expressed ATRAID using commercially available antibodies. All‐trans retinoic acid‐induced differentiation factor Iso C was seen in the vicinity of the plasma membrane, in the Golgi area, and in membranous compartments or vesicles in the cytosol, including lysosomes or endosomes as confirmed by the colocalization with LAMP1 and LAMP2. In addition, we found colocalization between ATRAID and RAB11, a GTPase associated with recycling endosomes (REs) and implicated in regulating vesicular trafficking.

## Materials and methods

### *In silico* analysis

Prediction of signal peptide and glycosylation sites was performed using signalp‐6.0 (https://services.healthtech.dtu.dk/service.php?SignalP) [13] and netnglyc‐1.0 (https://services.healthtech.dtu.dk/services/NetNGlyc‐1.0/) [14], respectively. Location of ATRAID in the cellular compartments was predicted using deeploc 2.0 (https://services.healthtech.dtu.dk/service.php?DeepLoc‐2.0) [15], a protein location prediction tool. Its structural features were examined using interpro (https://www.ebi.ac.uk/interpro/) [3] and prosite (https://prosite.expasy.org) [4, 16].

### Cell culturing

Human immortalized fibroblasts (Hum‐Fib) (Cat. No. CRL‐2522, ATCC), hTERT‐immortalized retinal pigment epithelial cell (RPE‐1) (Cat. No. CRL‐4000, ATCC), ARPE‐19 (Cat. No. CRL‐2302, ATCC), and human embryonic kidney cells 293 (HEK293) (Cat. No. CRL‐1573TM, ATCC) were cultured in Dulbecco's modified Eagle's medium high glucose with sodium pyruvate, without l‐Glutamine (Cat. No. ECB7501L, EuroClone, Pero, Italy), supplemented with 10% fetal bovine serum (FBS) (Cat. No. F7524‐500ML, Sigma‐Aldrich, Inc., St. Louis, MO, USA), penicillin 100 U·mL^−1^, streptomycin 100 μg·mL^−1^ (Cat. No. DE17‐602E, Lonza, Lonza bioscience, Walkersville, MD, USA), and 2 mm l‐glutamine (Cat. No. G7513, Sigma‐Aldrich, Inc.) at 37 °C with 5% CO_2_.

### Mammalian expression vectors

Mammalian expression vectors containing coding sequences of ATRAID isoforms with a Myc‐Flag tag at their C terminal end were purchased from Origene: NM_016085 (Cat. No. RC203440) (Iso A), NM_080592 (Cat. No. RC213998) (Iso B), and NM_001170795 (Cat. No. RC229723) (Iso C). Iso A and Iso C were subsequently subcloned into the retroviral vector pQCXIP (Cat. No. 631516, Takara Bio, San Jose, CA, USA). Briefly, subcloning was performed by PCR amplification of the complete cDNA, using the Origene plasmids as template and PCR primers with specific sites for restriction enzymes added to the 5′ end (BamH I and EcoR I) (see Table S1). Following PCR amplification, the products were incubated with Dpn I, then purified using the QIAGEN PCR Cleanup kit (Cat. No. 28106, QIAGEN, Hilden, Germany). Purified PCR products were digested by BamH I (Cat. No. R3136S, New England Biolabs, Ipswich, MA, USA) and EcoR I (Cat. No. R3101S, New England Biolabs). Similarly, the retroviral vector was cut with the same restriction enzymes and dephosphorylated by adding calf intestinal phosphatase (Cat. No M2825, Promega, Madison, WI, USA) and purified using the QIAGEN PCR Cleanup kit. Finally, ligation was performed using T4 DNA ligase (Cat. No. M0202M, New England Biolabs) with overnight incubation at 16 °C, according to the manufacturer's recommendations.

Transformation was carried out using XL10‐Gold Ultracompetent Cells (Cat. No. 200315, Agilent, Santa Clara, CA, USA), following the manufacturer's recommendations. Transformed cells were streaked on Invitrogen™ imMedia™ Amp Growth Medium (Cat. No. Q60120, Invitrogen) and incubated at 37 °C overnight. The next day, 5 mL Invitrogen™ imMedia™ Amp liquid Growth Medium (Cat. No. Q60020, Invitrogen) was inoculated with a single colony and incubated overnight on a shaker at 37 °C. Plasmid purification was performed using QIAprep Spin Miniprep Kit (Cat. No. 27106, QIAGEN). The OD 260/280 of the purified plasmid DNA was measured by Nanodrop (NanoDrop One, ThermoFisher Scientific, Waltham, MA, USA), and the sequence verified by Sanger sequencing.

### Transient transfection

HEK293 and ARPE‐19 cells were seeded in 60‐mm dishes and transfected at 60–70% confluency with Origene mammalian expression plasmids containing the coding sequence of Myc‐Flag tagged ATRAID corresponding to the three transcripts (Iso A, Iso B and Iso C), using Lipofectamine™ 3000 Transfection Reagent kit (Cat. No. L3000001, ThermoFisher Scientific), according to the manufacturer's protocol.

### Transduction

Phoenix‐AMPHO retroviral packaging cells (FXA) (Cat. No. CRL‐3213, ATCC) were transfected with the ATRAID‐pQCXIP by calcium phosphate precipitation [17] (see Appendix S1). The culture medium of the packaging cells containing the retroviral particles was collected and filtered 48 h post‐transfection. ARPE‐19, RPE‐1, Hum‐Fib, and HEK293 cells were transduced by overlaying media collected from the transfected packaging cells and incubated for 24 h. Stably transduced cells were subsequently selected in medium containing 1 μg·mL^−1^ puromycin (Cat. No. ant‐pr‐1, InvivoGen, Toulouse, France). Transduced cells were kept in selection medium for at least 6 days.

### Subcellular fractionation

Cells were seeded in 25‐cm^2^ flasks and grown until 70–80% confluency, washed with cold Dulbecco's phosphate‐buffered saline (PBS) (Cat. No. D8537, Sigma‐Aldrich), and detached in 3 mL of cold PBS with 10 mm EDTA (Cat. No. 15575‐038, Invitrogen). The suspended cells were washed twice with cold PBS and centrifuged for 3 min at 1300 ***g***. The final cell pellet was resuspended in 1 mL of cold homogenization buffer (10 mm HEPES pH 7.4, 50 mm sucrose, 1 mm PMSF, 1 μg·mL^−1^ aprotinin), supplemented with Complete™ Mini EDTA‐free Protease Inhibitor Cocktail (Cat. No. 11836170001, Sigma‐Aldrich). The cell suspensions of HEK293 and ARPE‐19 were homogenized with 40 and 35 strokes, respectively, using a cell homogenizer (Isobiotec, Heidelberg, Germany) with a 10‐μm clearance ball. The cell lysate was centrifuged at 2000 ***g*** for 10 min at 4 °C. The pellet, representing the nuclear fraction, was resuspended in 150 μL of homogenization buffer while the supernatant was centrifuged for another 60 min at 135 700 ***g*** at 4 °C (TLA100.2 rotor, Optima MAX‐XP ultracentrifuge, Beckman Coulter, Brea, CA, USA). The supernatant of this step represents the cytosolic fraction, and the pellet represents the membranous fraction. The membranous fraction was washed by resuspending the pellet in 1 mL of homogenization buffer and further centrifuged at 135 700 g at 4 °C for 15 min. The supernatant was discarded, and the pellet resuspended in 150 μL RIPA lysis and extraction buffer (Cat. No. 89900, ThermoFisher) including one tablet complete™ Mini EDTA‐free Protease Inhibitor Cocktail per 7 mL lysis buffer (Cat. No. 11836170001, Roche, Mannheim, Germany). Following the acetone precipitation, fractions were resuspended in 10 μL RIPA lysis and extraction buffer.

### Deglycosylation

Both O‐ and N‐deglycosylation of protein lysates were performed as previously described [18] (see Appendix S1). N‐deglycosylation of cells seeded on glass slides for immunofluorescence analysis was performed as described by Lee and coworkers [19] (see Appendix S1).

### Treatment with MG132 and leupeptin

Transduced cell lines expressing Myc‐Flag tagged ATRAID Iso A were seeded in 25‐cm^2^ tissue culture flasks. At 50% confluency, the cells were treated with either 10 μm MG132 (Cat.No.C2211, Sigma‐Aldrich), 10 μm leupeptin (Cat. No. 1167, TOCRIS, Bristol, UK), or DMSO (vehicle) only (Cat. No. D8418, Sigma‐Aldrich). After 5‐h incubation at 37 °C, the cells were lysed in RIPA lysis and extraction buffer and subjected to immunoblot analysis with anti‐Flag antibody.

### Immunoblot analysis

After the addition of Laemmli Sample Buffer (Cat. No. 1610747, Bio‐Rad) including NuPAGE™ Sample Reducing Agent (10X) (Cat. No, NP0009, Thermo Fisher), cell lysates were separated on 4–20% Mini‐PROTEAN TGX Stain‐Free™ protein gels (Cat. No. 4568095, Bio‐Rad, Hercules, CA, USA), and transferred to Trans‐Blot Turbo Mini 0.2 μm Nitrocellulose membranes (Cat. No. 1704158, Bio‐Rad). Membranes were blocked in 5 mL EveryBlot Blocking Buffer (c#. 12010020, Bio‐Rad) for 30 min. Incubation with primary antibodies diluted in blocking buffer, including rabbit anti‐APR3 (Cat. No. PA5‐62388, ThermoFisher, 1/1000 dilution), rabbit anti‐Flag (Cat. No. PA1‐984B, ThermoFisher, 1/1000 dilution), and rabbit anti‐GAPDH (Cat. No. G9545, Sigma‐Aldrich, 1/5000 dilution) was performed over night at 4 °C. Membranes were washed 3 × 5 min in Tris‐buffered saline (TBS) pH 7.6 with 0.1% Tween 20, then incubated with HRP‐linked anti‐rabbit IgG secondary antibodies (Cat. No. 7074, Cell Signaling Technology, 1/2000 dilution) for 1 h at room temperature and washed 3 × 5 min. Membranes were developed using the Clarity Max Western ECL Substrate (Cat. No. 1705062, Bio‐Rad) or Clarity Western ECL Substrate (Cat. No. 1705061, Bio‐Rad), and scanned on the ChemiDocTM MP (Bio‐Rad) Imaging System.

### Immunofluorescence analysis

Approximately 50 000 cells were seeded in 12‐well plates on 12‐mm circular cover slips (Cat. No. 117520, Marienfeld, Lauda‐Königshofen, Germany). At 60–70% confluency, immunofluorescence analysis was performed according to Sannerud and coworkers [20] (see Appendix S1), except that all incubations with primary antibodies were performed overnight at 4 °C. Deglycosylation of cells was performed in all experiments where endogenous ATRAID was detected using anti‐APR3 antibodies. Prior to RAB11 antibody incubation, target retrieval was performed using 6 M guanidine‐HCl as previously described [21] (see Appendix S1).

Primary antibodies were rabbit anti‐Flag (Cat. No. PA1‐984B, ThermoFisher, 1/1000 dilution), mouse anti‐Flag (Cat. No. F1804‐UG50, Sigma‐Aldrich, 1/500 dilution), rabbit anti‐APR3 (Cat. No. PA5‐62388, ThermoFisher, 1/50 dilution), rabbit anti‐GALNT2 (Cat. No. HPA011222, Sigma‐Aldrich, 1/200 dilution), mouse anti‐RAB11 (Cat. No. 610657, BD Biosciences, Franklin Lakes, NJ, USA, 1/50 or 1/100 dilution), mouse anti‐LAMP1 (Cat. No. 555798, BD Biosciences, 1/50 dilution), mouse anti‐LAMP2 (Cat. No. H4B4, DSHB, 1/50 dilution), mouse anti‐GM130 (Cat. No. 610823, BD Biosciences, 1/20 dilution), and mouse anti‐TOMM20 (Cat. No. WH0009804M1, Sigma‐Aldrich, 1/25 dilution).

Secondary antibodies were Alexa Fluor® 488‐conjugated Affinity Purified F(ab′)2 Fragment Goat anti‐Mouse IgG (H + L) (Cat.No. 115‐546‐062, Jackson ImmunoResearch, Cambridge, UK), Alexa Fluor® 594‐conjugated Affinity Purified F(ab′)2 Fragment Goat anti‐Mouse IgG (H + L) (Cat. No. 115‐586‐146, Jackson ImmunoResearch), Alexa Fluor® 488‐conjugated Affinity Purified F(ab′)2 Fragment Goat anti‐Rabbit IgG (H + L) (Cat. No. 111‐546‐144, Jackson ImmunoResearch), Alexa Fluor® 594‐conjugated Affinity Purified F(ab′)2 Fragment Goat anti‐Rabbit IgG (H + L) Cat. No. 111‐586‐144, Jackson ImmunoResearch) diluted 1/200 (see Appendix S1).

## Results

### *In silico* predictions

Although bioinformatic analysis of the ATRAID protein has been reported previously [2], the software capabilities have evolved since then and the predictions have changed somewhat. The genomic structure of the three ATRAID transcripts (Iso A, Iso B, and Iso C) is illustrated in Fig. S1. When using the genomic sequence as a reference, it is evident that the coding regions of the predicted isoforms of ATRAID all share the last five exons. Iso A is using an internal start codon within exon 2 and has a different sequence in the 5′UTR compared with the two other transcripts. Iso B is the largest transcript, but the difference between Iso B and Iso C is restricted to sequences in the 5′ and 3′ UTR's (10 nt in the 5′UTR and 41 nt in the 3′UTR of Iso B as compared to Iso C). The predicted start codon of Iso B is upstream the one used by Iso C, although apart from minor differences in UTR's the sequences are identical. Hence, the difference between the three postulated isoforms can be explained by utilizing different start codons. Signal peptide prediction using SignalP‐6.0 on ATRAID Iso C (NP_001164266.1) reported with high confidence a potential N‐terminal signal peptide within the first 30 aa, directly followed by a peptidase cleavage site located between aa 30 and 31 [13] (Fig. S2). No signal sequence was predicted for Iso A or B. Several N‐glycosylation sites were predicted by NetNGlyc‐1.0, located at aa position 44, 79, 157, and 168 of Iso C [14]. DeepLoc 2.0, a protein localization prediction tool, predicted ATRAID Iso C to be located at the cell membrane (probability = 0.78), the lysosome/vacuole (probability = 0.60), and to the Golgi apparatus (probability = 0.32) [15]. Based on InterPro and PROSITE, ATRAID contains several predicted functional domains, including an N‐terminal signal sequence, a Toll‐like leucine‐rich repeat followed by an EGF‐domain, a single transmembrane region and a short cytoplasmic C‐terminal part [3, 4, 16] (Fig. S2).

### Specificity of antibodies

For detection and visualization of ATRAID, we initially tested several commercially available antibodies, using as positive control either immunofluorescence analysis of transduced cells expressing Flag‐tagged ATRAID or immunoblot analysis of cell extracts from transduced cells before and after deglycosylation.

**Fig. 1:**
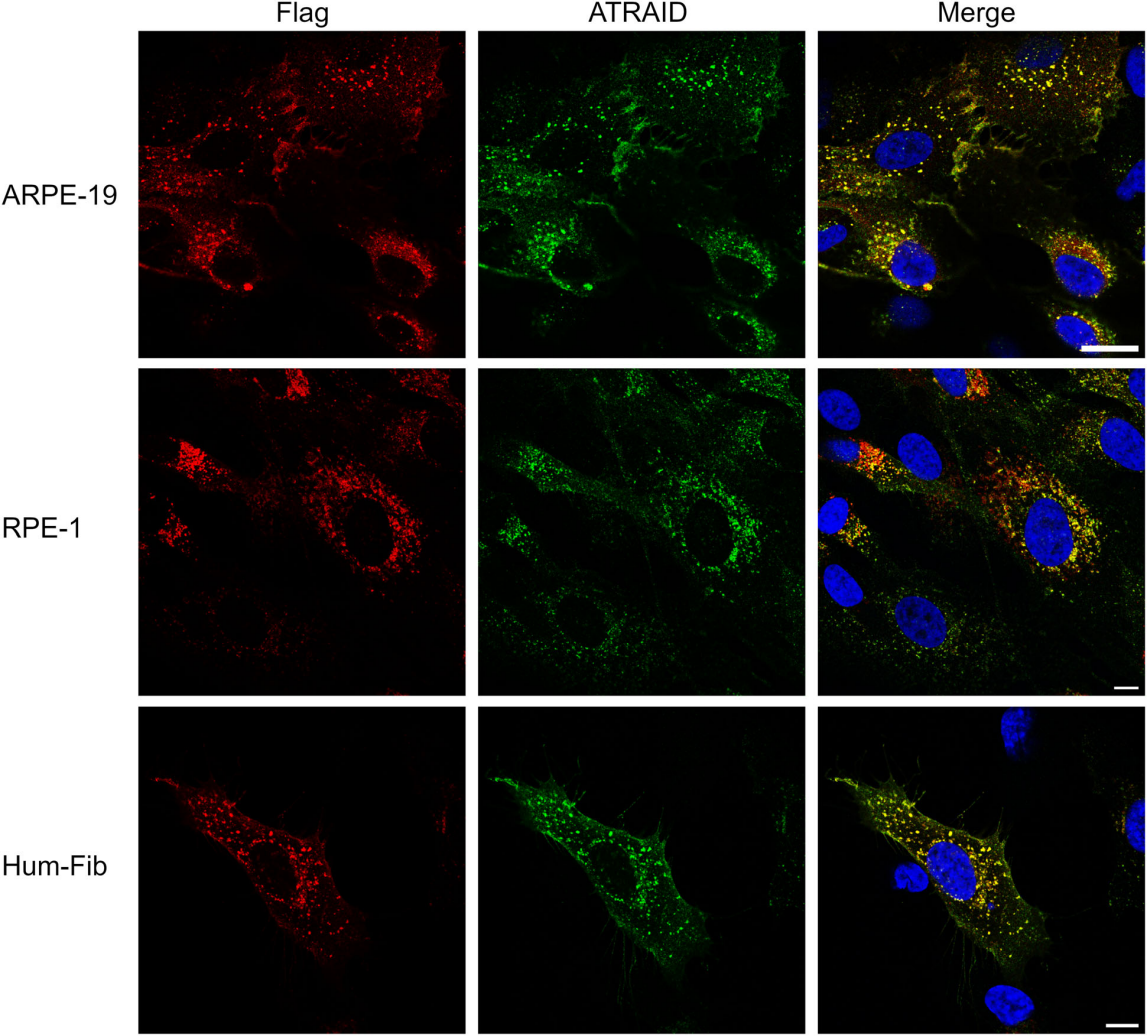
Immunofluorescence analysis with anti‐Flag (red) and anti‐ATRAID (green) antibodies in three transduced cell lines expressing Myc‐Flag‐tagged ATRAID Iso C. Merged images show complete colocalization between Flag and ATRAID antibodies. Blue is DAPI staining. Scale bar: 10 μm.

Among the commercial anti‐ATRAID antibodies, we found two that worked well in immunoblot analysis following the deglycosylation of cell lysates (data not shown). For further applications, we selected the one showing the highest sensitivity (Cat. No. PA5‐62388, ThermoFisher). To examine the specificity of the antibody, double labeling immunofluorescence analysis was performed on transduced cell lines expressing Myc‐Flag‐tagged ATRAID Iso C, using mouse anti‐Flag and rabbit anti‐ATRAID antibodies. We observed a complete overlap of the staining produced by the two antibodies (Fig. 1).

### ATRAID protein isoforms

**Fig. 2:**
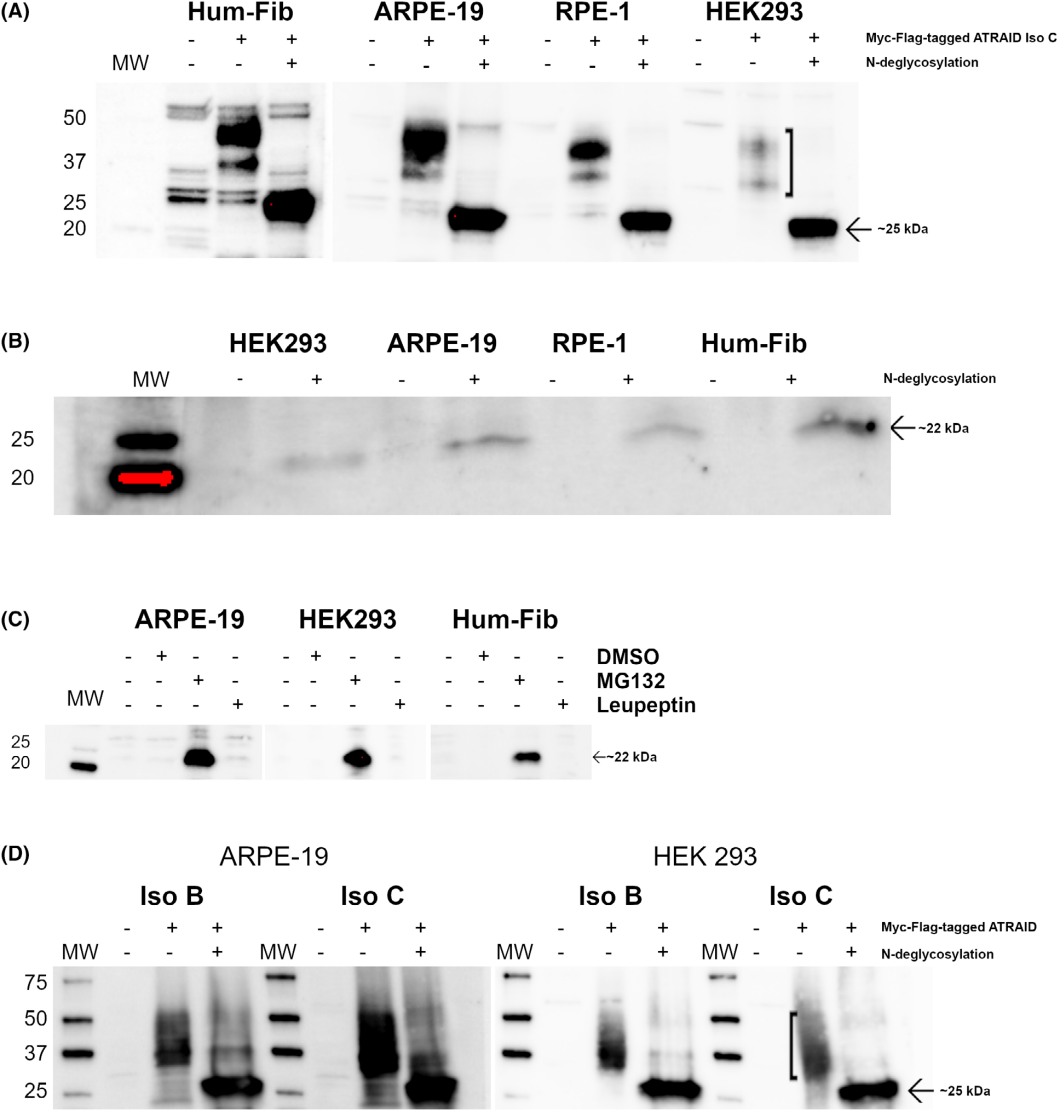
Immunoblot analysis of ATRAID isoforms. (A) Lysates from transduced cells expressing Myc‐Flag‐tagged Iso C, before and after N‐deglycosylation, incubated with anti‐Flag antibodies. Arrow: 25.2 kDa, Box bracket: Smears of glycosylated protein. (B) Endogenous expression: Lysates from different cell lines before and after N‐deglycosylation incubated with antibodies against ATRAID. Arrow: 21.6 kDa. (C) Lysates from transduced cells expressing Myc‐Flag‐tagged Iso A incubated with anti‐Flag antibodies before and after treatment with vehicle (DMSO), proteasome inhibitor MG‐132 or lysosomal inhibitor Leupeptin. Arrow: 21.8 kDa. (D) Lysates from cells transfected with plasmids expressing either Myc‐Flag‐tagged Iso B or Iso C before and after N‐deglycosylation incubated with anti‐Flag antibodies. Arrow: 25.2 kDa, Box bracket: smears of glycosylated protein (whole blots are presented in Fig. S5).

Immunoblot analysis using anti‐Flag antibodies on cell lysates from transduced cells expressing Myc‐Flag tagged ATRAID Iso C revealed that the expressed protein migrated as a smear, indicating that the protein undergoes some type of post‐translational modification (Fig. 2A). Following N‐deglycosylation, a distinct band with the expected molecular weight (MW) of approximately 25 kDa appeared consistent with a cleaved signal peptide. The MW of the complete protein is estimated to be 24.7 kDa with the theoretical MW of the signal peptide being 3.1 kDa. The MW of the Myc‐Flag tag is approximately 3.6 kDa. O‐deglycosylation did not result in any obvious change in the migration of Myc‐Flag‐tagged ATRAID Iso C (Fig. S3).

Lysates from different cell lines were examined by immunoblot analysis for endogenously expressed ATRAID. Following N‐deglycosylation, a band with relative MW of approximately 21–22 kDa appeared, again consistent with the signal peptide being cleaved off (Fig. 2B).

In transduced cells expressing Myc‐Flag‐tagged ATRAID Iso A, the protein was not detectable using the anti‐Flag antibody unless treated with the proteasome inhibitor MG132. Following the MG132 treatment, a band corresponding to Myc‐Flag‐tagged Iso A with the expected size, 21.8 kDa, appeared (Fig. 2C). Treatment with the lysosomal inhibitor, Leupeptin, did not show any effect. No indication of post‐translational modification of Iso A was observed (Fig. S4).

Transient transfection of HEK293 and ARPE‐19 cells with mammalian expression vectors containing Myc‐Flag tagged Iso B or Iso C, followed by immunoblotting of cell lysates, showed that both transcripts produced the same protein isoform. Both were highly N‐glycosylated and, when deglycosylated, produced a protein of the same molecular weight of approximately 25 kDa (Fig. 2D). The postulated Iso B protein (284 aa, estimated MW 30.3 kDa, UniProt Q6UW56‐3) was not detected.

### Intracellular localization of ATRAID Iso C

**Fig. 3:**
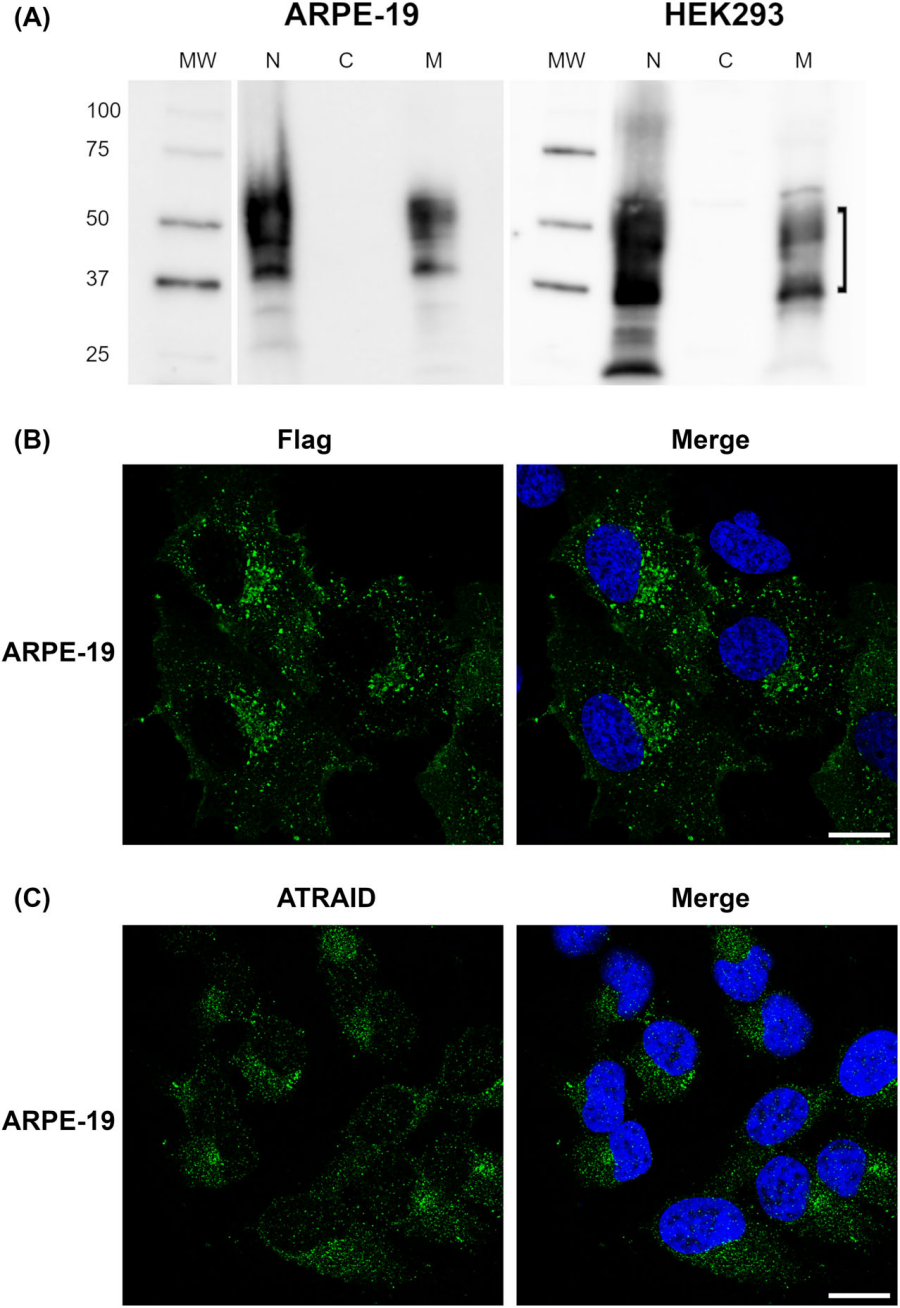
Intracellular localization of ATRAID. (A) Immunoblot analysis of transduced ARPE‐19 and HEK293 cells overexpressing Myc‐Flag tagged ATRAID Iso C after subcellular fractionation using anti‐Flag antibodies. Box bracket: Smears of glycosylated protein. N—nuclear fraction, C—cytosolic fraction, M—membranous fraction, MW: MW standard (kDa) (whole blot is presented in Fig. S5). (B) Exogenous expression: Immunofluorescence analysis of transduced ARPE‐19 cells overexpressing Myc‐Flag‐tagged ATRAID Iso C using anti‐Flag antibodies. (C) Endogenous expression: Immunofluorescence analysis of deglycosylated wild‐type ARPE‐19 cells with anti‐ATRAID antibodies. Blue is DAPI staining. Scale bar: 10 μm.

Following subcellular fractionation of transduced cells expressing Myc‐Flag‐tagged ATRAID Iso C, immunoblot analysis using anti‐Flag antibody showed that the protein was localized in the nuclear and membranous fractions, while being undetectable in the cytosolic fraction (Fig. 3A). Immunofluorescence staining of transduced cells expressing Myc‐Flag tagged ATRAID Iso C with anti‐Flag antibody showed ATRAID Iso C in vesicle‐like structures throughout the cytoplasm, in areas/compartments close to the nucleus and in the vicinity of the plasma membrane (Fig. 3B). In nontransduced cells, endogenous ATRAID expression was seen mainly in compartments close to the nucleus (Fig. 3C). Although the endogenous expression is much lower, the pattern clearly resembles that observed with anti‐Flag antibody in transduced cells.

### ATRAID Iso C colocalizes with LAMP1, LAMP2, GALNT2/GM130 and RAB11

**Fig. 4:**
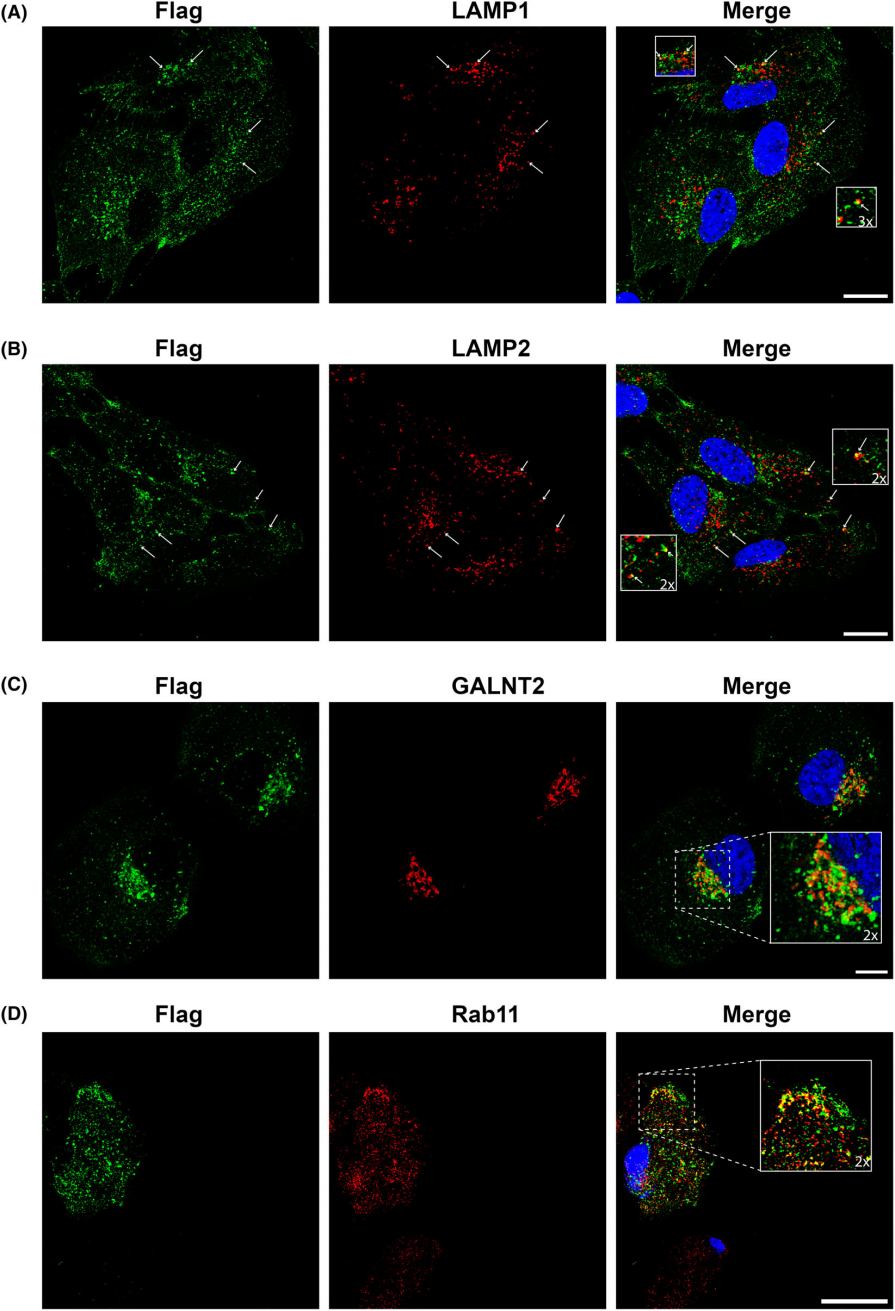
Immunofluorescence analysis of transduced ARPE‐19 cells overexpressing Myc‐Flag‐tagged ATRAID Iso C (exogenous expression). (A) Costaining with anti‐Flag and anti‐LAMP1 antibodies. (B) Costaining with anti‐Flag and anti‐LAMP2 antibodies. (C) Costaining with anti‐Flag and anti‐GALNT2 antibodies. (D) Costaining with anti‐Flag and anti‐Rab11 antibodies. Blue is DAPI staining. Arrows and boxes indicate colocalization. Scale bar: 10 μm.

For colocalization experiments, we initially performed double staining immunofluorescence analysis of transduced cells expressing Myc‐Flag tagged ATRAID Iso C. Antibodies against both the lysosomal/endosomal markers LAMP1 and LAMP2 reacted with vacuole‐like structures in a similar pattern. Some of the Myc‐Flag‐tagged ATRAID Iso C colocalized with LAMP1 and to some extent with LAMP2 (Fig. 4A,B, Fig. S6). However, ATRAID was also found to be present in other membranous structures. Antibodies against the Golgi marker GALNT2 revealed ATRAID in the Golgi area (Fig. 4C, Fig. S7A). We also examined a marker for endosomes, RAB11, which is found primarily in recycling endosomes. Colocalization of ATRAID and RAB11 was evident mainly in protrusions at the periphery of the cell close to the plasma membrane (Fig. 4D, Fig. S7B). RAB11 is known to participate in the formation of the pericentrosomal endocytic recycling compartment (ERC). In some cells, colocalization of ATRAID with RAB11 was seen in the area of the ERC. No colocalization was seen with the mitochondrial marker TOMM20 (Fig. S8).

**Fig. 5:**
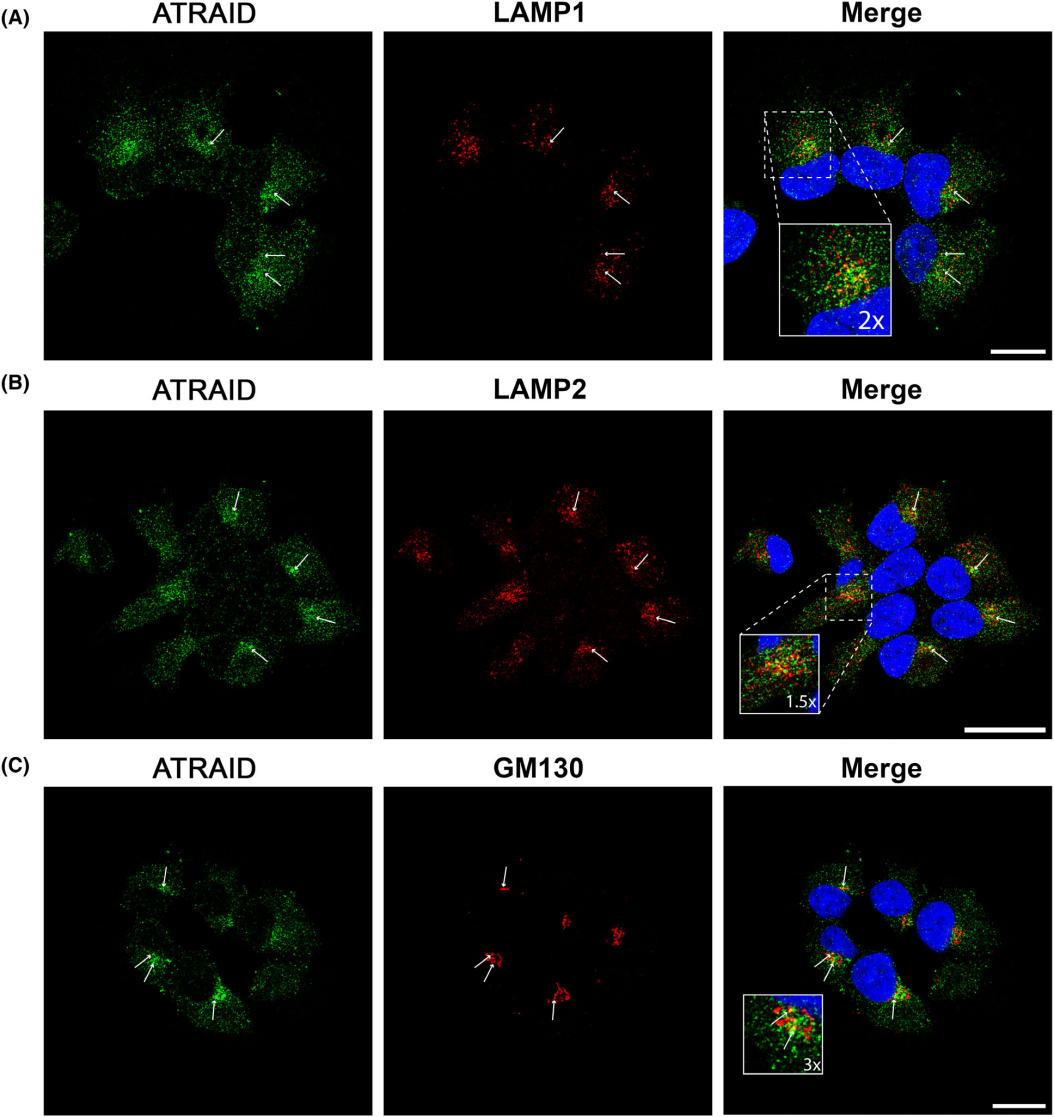
Immunofluorescence analysis of deglycosylated ARPE‐19 cells (endogenous expression). (A) Costaining with anti‐ATRAID and anti‐LAMP1 antibodies. (B) Costaining with anti‐ATRAID and anti‐LAMP2 antibodies. (C) Costaining with anti‐ATRAID and anti‐GM130 antibodies. Blue is DAPI staining. Arrows and boxes indicate colocalization. Scale bar: 10 μm.

Endogenous ATRAID colocalized with LAMP1, LAMP2 (Fig. 5A,B), and with the Golgi marker GM130 (Fig. 5C). GALNT2 was replaced with GM130 since anti‐GALNT2 and anti‐APR‐3 antibodies were raised in the same species. We were unable to examine colocalization between endogenous ATRAID and RAB11. The detection of RAB11 required target retrieval using a 6 M guanidine‐HCl buffer. Unfortunately, this procedure was not tolerated by the anti‐APR3 antibody.

## Discussion

All‐trans retinoic acid‐induced differentiation factor was first identified among differentially expressed genes after all‐trans retinoic acid stimulation of HL‐60 genes and named APR3 [1]. Shortly thereafter, it was recognized in a cDNA library derived from CD34+ hematopoietic stem/progenitor cells (HSPCs) and named HSPC013 [22]. Various isoforms of ATRAID have been used in previous studies, most often Iso B [5, 6, 7, 8, 10, 23, 24]. In NCBI, three RefSeq transcripts of ATRAID can be found, NM_016085 (Iso A), NM_080592 (Iso B), and NM_001170795 (Iso C). NCBI recently retracted NM_080592 (Iso B) since there was support only for the transcript, but not for the protein. The difference between the Iso B and Iso C transcript is a 10 nt extension of the 5′UTR and 41 nt of the 3′UTR (in Iso B compared to Iso C). Other than that, the sequences are identical. When we examined the expression of ATRAID, both endogenously and in transduced or transfected cells, we found Iso C as the main detectable form. Iso A is expressed in transduced cells but is rapidly degraded by proteasomes. No indication of post‐translational modification of Iso A was observed (Fig. S4). In cells transiently transfected with the coding region of Iso B, we did not see the postulated Iso B protein. However, we did observe the expression of Iso C (Fig. 2D). Considering the genomic structure and the proposed coding region of the three ATRAID isoforms, it is evident that they all share the same reading frame and can be explained by differential use of initiation codons (Fig. S1). Sharing the C‐terminal part, the notable difference can be found in the N‐terminal part. In isoform C, there is a predicted N‐terminal signal peptide, possibly directing it to the ER/Golgi for post‐translational modification. The signal peptide is absent in Iso A, as Iso A is initiated from a downstream initiation codon, also the sequence of the 5′UTR of IsoA differ from the other two transcripts. As Iso A is lacking a signal peptide, it is not transported to the ER/Golgi and consequently appears unglycosylated. Likewise, the postulated Iso B protein would lack this N‐terminal signal peptide as it would be surpassed by 55 aa in the N‐terminal part and consequently not be a functional signal peptide. No signal sequence was predicted for the Iso B translated isoform by SignalP. For the same reasons as for Iso A described above, translation of Iso B would not lead to a glycosylated protein product. We therefore conclude that translation of Iso B and Iso C initiates at the same initiation codon. As this is shared by both postulated isoforms (Iso B and Iso C), translation of both transcripts results in one and the same protein (NP_001164266).

In immunoblot analysis, we found that Myc‐Flag‐tagged ATRAID Iso C migrated as a smear. After N‐deglycosylation, a single band corresponding to a MW of approximately 25 kDa was observed (Fig. 2A). The MW is in concordance with that of the full‐length protein with the Myc‐Flag tag after the signal peptide has been cleaved off (28.3 kDa minus 3.1 kDa). We were not able to detect endogenously expressed ATRAID in untreated cell extracts. However, after N‐deglycosylation, we observed a single, well‐defined band with an apparent MW around 21–22 kDa. Again, this result corresponds with the theoretical MW of endogenously expressed ATRAID Iso C where the signal peptide has been cleaved off (Fig. 2B). By performing double labeling experiments, we tested the specificity of commercial ATRAID antibodies. After deglycosylation, a complete overlap between anti‐Flag and anti‐ATRAID antibodies was observed, confirming the specificity of the anti‐ATRAID antibodies (Fig. 1). It is likely that the glycosylation masks the epitope of the commercial antibodies as they were produced by immunization with synthetic peptides. All in all, we conclude that the main endogenously expressed ATRAID isoform is Iso C, the predicted signal peptide is cleaved, and the protein is further post‐translationally modified by N‐glycosylation.

By combining subcellular fractionation and immunofluorescence analysis, we found that Myc‐Flag‐tagged ATRAID Iso C localized to membranous compartments or vesicles in the cytoplasm with higher density in areas close to the nucleus. In subcellular fractionation experiments, we observed Myc‐Flag‐tagged ATRAID Iso C both in the membranous and in the nuclear fractions but were unable to detect it in the nucleus by immunofluorescence analysis. The presence of ATRAID in the nuclear fraction is most likely due to membranes accompanying nuclei during centrifugation [25].

Previous studies of intracellular localization of ATRAID have been performed using transfected cells. Endogenous ATRAID expression is relatively low and validated commercially available antibodies are difficult to find. Yu and coworkers observed N‐terminal HA‐tagged ATRAID in the plasma membrane in transfected MCF‐7 cells [5], while Zou and coworkers found ATRAID both in the envelope of the nucleus and in the perinuclear region of transfected SaoS2 and U2OS cells [6]. The cell lines and ATRAID constructs used in these studies were different from ours, which could explain the divergent observations.

In transfection experiments with N‐terminal‐tagged ATRAID Iso B, Li and coworkers observed that ATRAID interacted with NRF2 and KEAP1 in the mitochondria of ARPE‐19 cells [10]. In transduced cell lines expressing C‐terminal Myc‐Flag‐tagged ATRAID Iso C, we did not observe any colocalization between ATRAID and the mitochondrial protein TOMM20 (Fig. S8). Again, the reason for the discrepancy could be the different design of the constructs used for transfection/transduction. For ATRAID, we favor constructs with a C‐terminal tag to avoid disturbing the function of the signal peptide.

We do recognize the possible pitfalls using epitope‐tagged exogenously expressed proteins. However, C‐terminal epitope‐tagged isoforms of ATRAID have been shown to functionally rescue the cytosolic entry of NBPs in ATRAID −/− cells [7], suggesting that C‐terminal tagging does not necessarily lead to a dysfunctional protein. Also, we find the distribution of endogenous and exogenous ATRAID similar, although endogenous expression is lower and the relative distribution is more focused in the juxta‐nuclear area.

Several authors have located ATRAID to the lysosome [2, 7, 8, 9]. We were able to reproduce the colocalization between ATRAID and LAMP1 and LAMP2, but the majority of ATRAID‐positive structures did not react with LAMP1 or LAMP2. Since LAMP1 can be found both in lysosomes and late endosomes [26], some of the vesicles where ATRAID shows colocalization with LAMP1 could represent late endosomes.

We did find ATRAID in the Golgi area as seen from double staining with the Golgi marker GALNT2, which is consistent with its N‐glycosylation modification. We also observed that ATRAID colocalizes with RAB11. RAB11 is a marker for recycling endosomes and regulates vesicle transport via the endocytic recycling compartment and early endosomes to the trans‐Golgi network and the plasma membrane [26, 27]. In addition to its role in post‐translational modification of newly synthesized polypeptide chains, the Golgi apparatus is involved in intracellular trafficking, where proteins from recycling endosomes may reach the Golgi‐assisted, among others, by RAB11 [28]. In that respect, it is worth mentioning that the cellular localization where colocalization of ATRAID and RAB11 takes place is distinct compared with the localization we find colocalization between ATRAID and both LAMP1 and LAMP2. RAB11 has also been found to interact with the HIV‐1 Nef protein in a split‐ubiquitin‐based yeast two‐hybrid screen [12]. It was shown that Nef reroutes the immune costimulatory proteins CD80 and CD86 from the plasma membrane to the Golgi through endocytic vesicles that require the recruitment of RAB11 [29].

In summary, we find that the main ATRAID isoform expressed is Iso C. This is a highly N‐glycosylated protein that is present in lysosomes or endosomes, the Golgi area, as well as in vesicular structures throughout the cytoplasmic space. We confirm the colocalization between ATRAID and LAMP1 and here report co‐localization between both LAMP2 and with RAB11. The latter findings may facilitate further experiments elucidating the cellular roles of this protein.

## Conflict of interest

We confirm that the work herein has been carried out in accordance with Uniform Requirements for manuscripts submitted to Biomedical journals (http://www.icmje.org). None of the authors have any disclosure of conflicts of interest.

### Peer review

The peer review history for this article is available at https://www.webofscience.com/api/gateway/wos/peer‐review/10.1002/2211‐5463.13685.

## Author contributions

CB, ER, and OB conceived and supervised the study. RM and OB conducted the experiments. OB conducted bioinformatic analysis. RM, ER, and OB wrote the manuscript. RM, ER, CB, IC, and OB analyzed and reviewed the manuscript.

## Supporting information


**Fig. S1.** Schematic representation of human ATRAID mRNA transcript NM_080592, NM_001170795, and NM_016085 drawn to scale.
**Fig. S2.** Schematic representation of the three predicted human ATRAID protein isoforms drawn to scale.
**Fig. S3.** Immunoblot analysis of transduced ARPE‐19 overexpressing Myc‐Flag‐tagged ATRAID Iso C before and after O‐deglycosylation using anti‐Flag antibodies.
**Fig. S4.** Immunoblot analysis of lysates from cells transfected with plasmids expressing Myc‐Flag‐tagged Iso A before and after N‐deglycosylation using anti‐Flag antibodies.
**Fig. S5.** Complete whole blots corresponding to Figs 2 and 3.
**Fig. S6.** Immunofluorescence analysis of transduced RPE‐1 and Human fibroblasts (Hum‐Fib) cells overexpressing Myc‐Flag‐tagged ATRAID Iso C (complementing Fig. 4).
**Fig. S7.** Immunofluorescence analysis of transduced RPE‐1 and Human fibroblasts (Hum‐Fib) cells overexpressing Myc‐Flag‐tagged ATRAID Iso C (complementing Fig. 4).
**Fig. S8.** Immunofluorescence analysis of transduced ARPE‐19, RPE‐1 and Hum‐Fib cells overexpressing Myc‐Flag‐tagged ATRAID Iso C.
**Table S1.** DNA sequence of PCR primers for subcloning ATRAID Iso A and Iso C from Origene expression vectors to the murine retroviral vector pQCXIP.

## Data Availability

The supporting data for the finding of this study are available in the supplementary material of this article.
