# Supplementary material for: Functional characterization of all‐trans retinoic acid‐induced differentiation factor (ATRAID)
Source: FEBS Open Bio. 2023 Sep 7;13(10):1874–86. doi: 10.1002/2211-5463.13685 (PMC10549228; doi:10.1002/2211-5463.13685)
Supplement: Supplementary file 1 — Fig. S1. Schematic representation of human ATRAID mRNA transcript NM_080592, NM_001170795, and NM_016085 drawn to scale. Fig. S2. Schematic representation of the three predicted human ATRAID protein isoforms drawn to scale. Fig. S3. Immunoblot analysis of transduced ARPE‐19 overexpressing Myc‐Flag‐tagged ATRAID Iso C before and after O‐deglycosylation using anti‐Flag antibodies. Fig. S4. Immunoblot analysis of lysates from cells transfected with plasmids expressing Myc‐Flag‐tagged Iso A before and after N‐deglycosylation using anti‐Flag antibodies. Fig. S5. Complete whole blots corresponding to Figs 2 and 3. Fig. S6. Immunofluorescence analysis of transduced RPE‐1 and Human fibroblasts (Hum‐Fib) cells overexpressing Myc‐Flag‐tagged ATRAID Iso C (complementing Fig. 4). Fig. S7. Immunofluorescence analysis of transduced RPE‐1 and Human fibroblasts (Hum‐Fib) cells overexpressing Myc‐Flag‐tagged ATRAID Iso C (complementing Fig. 4). Fig. S8. Immunofluorescence analysis of transduced ARPE‐19, RPE‐1 and Hum‐Fib cells overexpressing Myc‐Flag‐tagged ATRAID Iso C. Table S1. DNA sequence of PCR primers for subcloning ATRAID Iso A and Iso C from Origene expression vectors to the murine retroviral vector pQCXIP. [file FEB4-13-1874-s001.pdf]

Supplementary figure S1

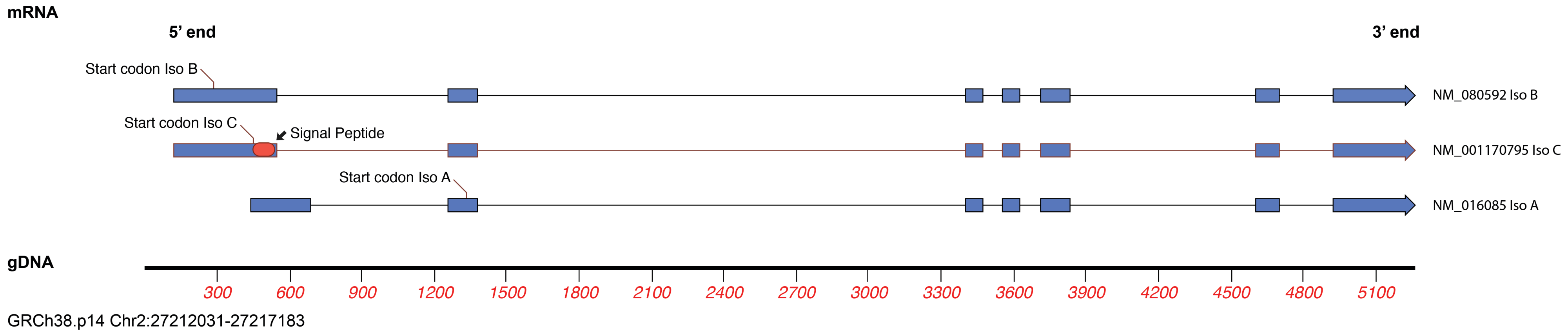

Schematic representation of human ATRAID mRNA transcript NM_080592, NM_001170795 and NM_016085 drawn to scale. Blue boxes are exons, including UTR's. Black line between boxes is introns. Location of start codon (ATG) for the three transcripts marked with line and text. Sequence translated to a predicted signal peptide marked in red.

Supplementary figure S2

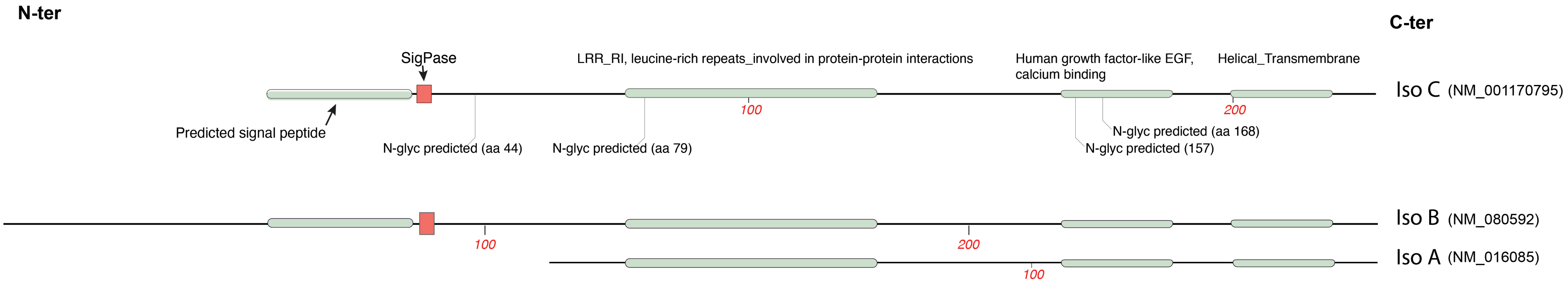

Schematic representation of the three predicted human ATRAID protein isoforms drawn to scale. N-terminal and C-terminal end indicated (N-ter and C-ter). Location of the four postulated N-glycosylation sites indicated with line and text. Yellow rounded boxes indicate predicted functional domains, including the predicted signal peptide in Iso C. SigPase indicate amino acid sequence recognized by peptidase.

Supplementary figure S3

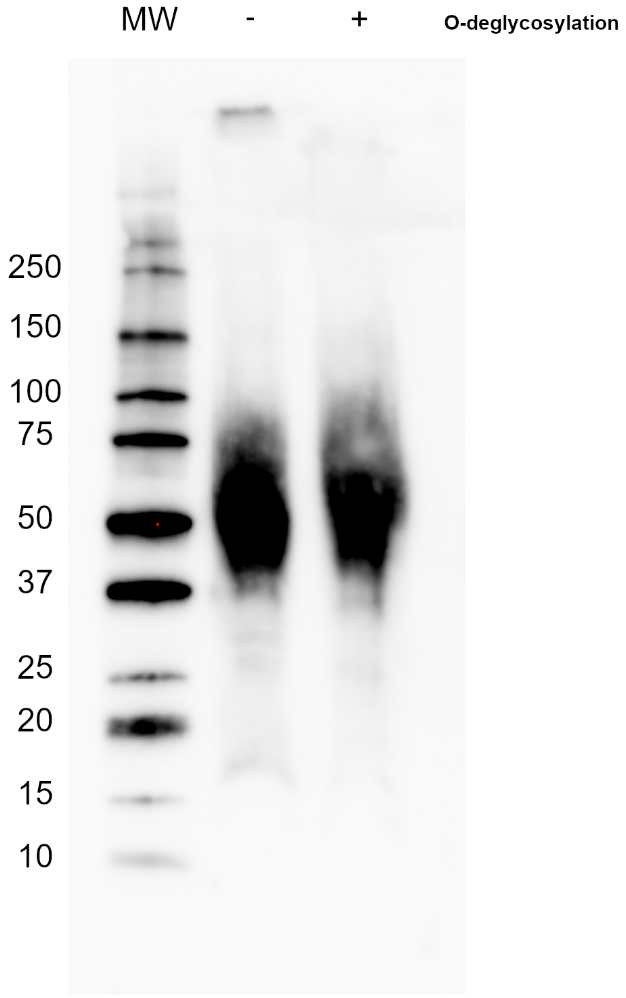

Immunoblot analysis of transduced ARPE-19 overexpressing Myc-Flag tagged ATRAID Iso C before and after O-deglycosylation using anti-Flag antibodies. O-deglycosylation did not have any effect on the migration of the protein.

Supplementary figure S4

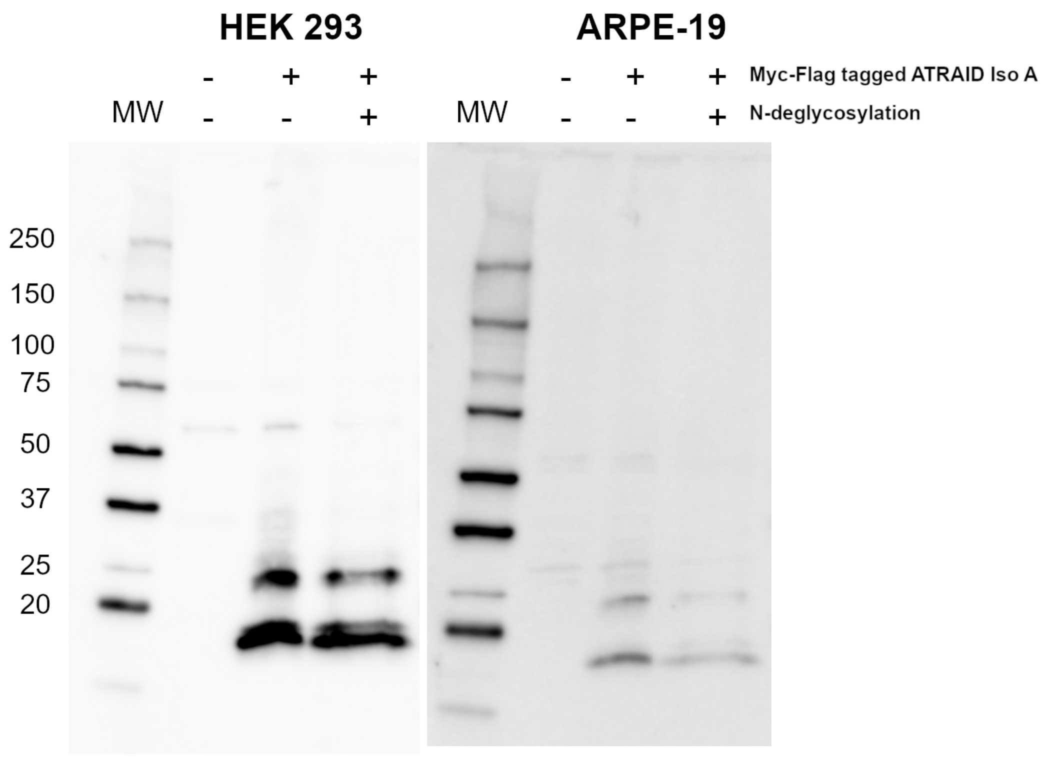

Immunoblot analysis of lysates from cells transfected with plasmids expressing Myc-Flag-tagged Iso A before and after N-deglycosylation using anti-Flag antibodies. N-deglycosylation did not have any effect on the migration of the protein.

Supplementary figure S5

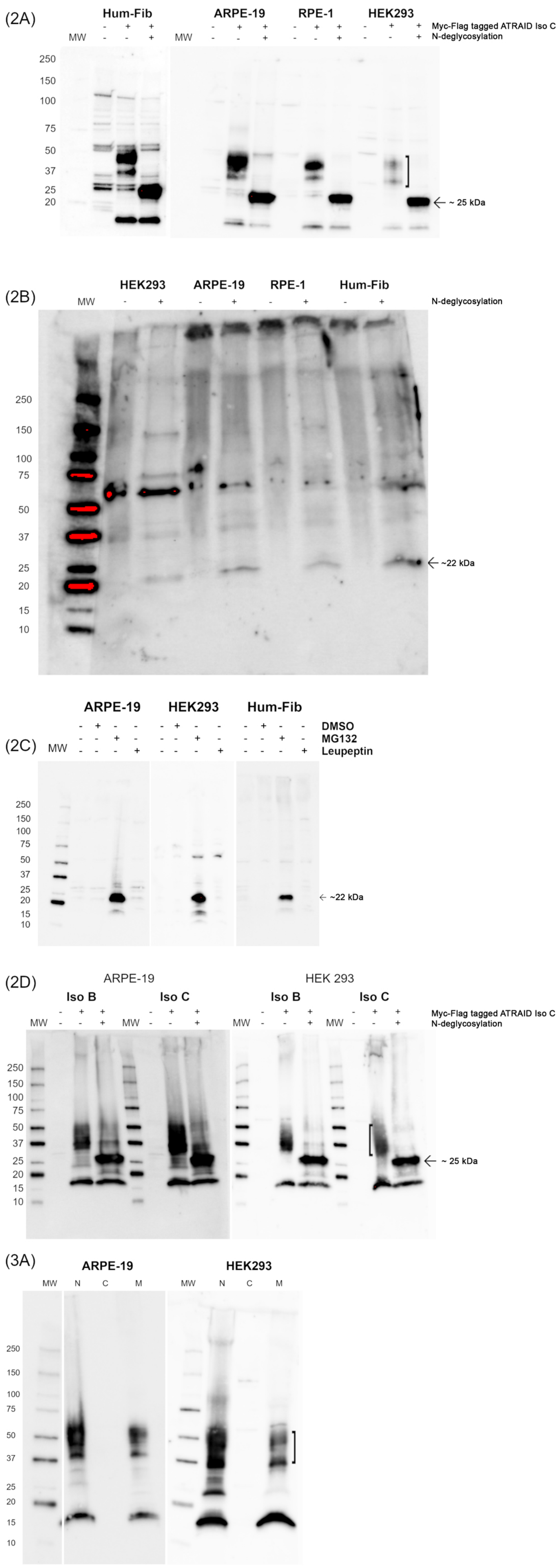

Complete whole blots corresponding to Figures 2 and 3.

Supplementary figure S6

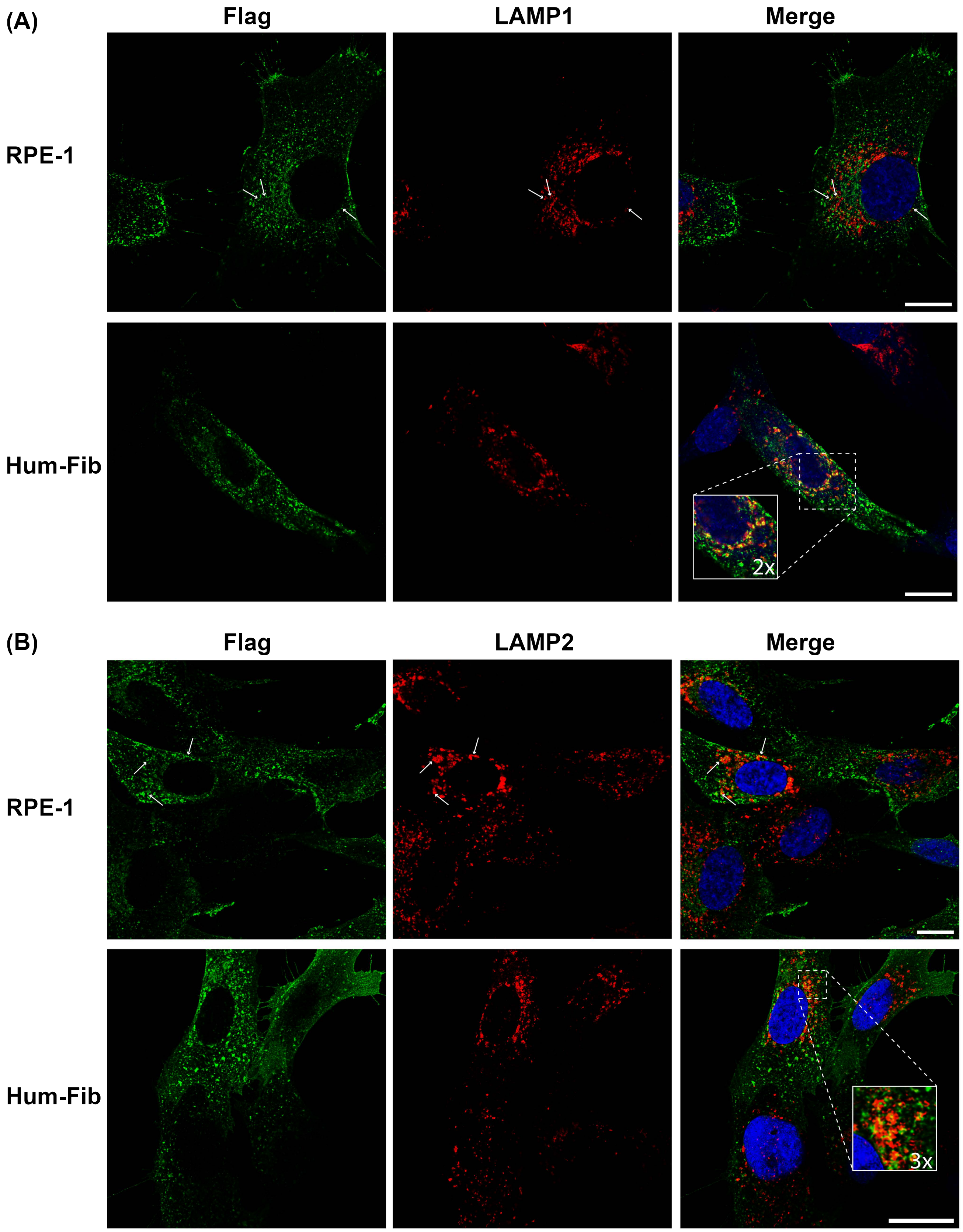

Immunofluorescence analysis of transduced RPE-1 and Human fibroblasts (Hum-Fib) cells overexpressing Myc-Flag-tagged ATRAID Iso C (complementing Figure 4). (A) Co-staining with anti-Flag and anti-LAMP1 antibodies. (B) Co-staining with anti-Flag and anti-LAMP2 antibodies. Blue is DAPI staining. Arrows and boxes indicate colocalization. Scale bar: 10 μm.

Supplementary figure S7

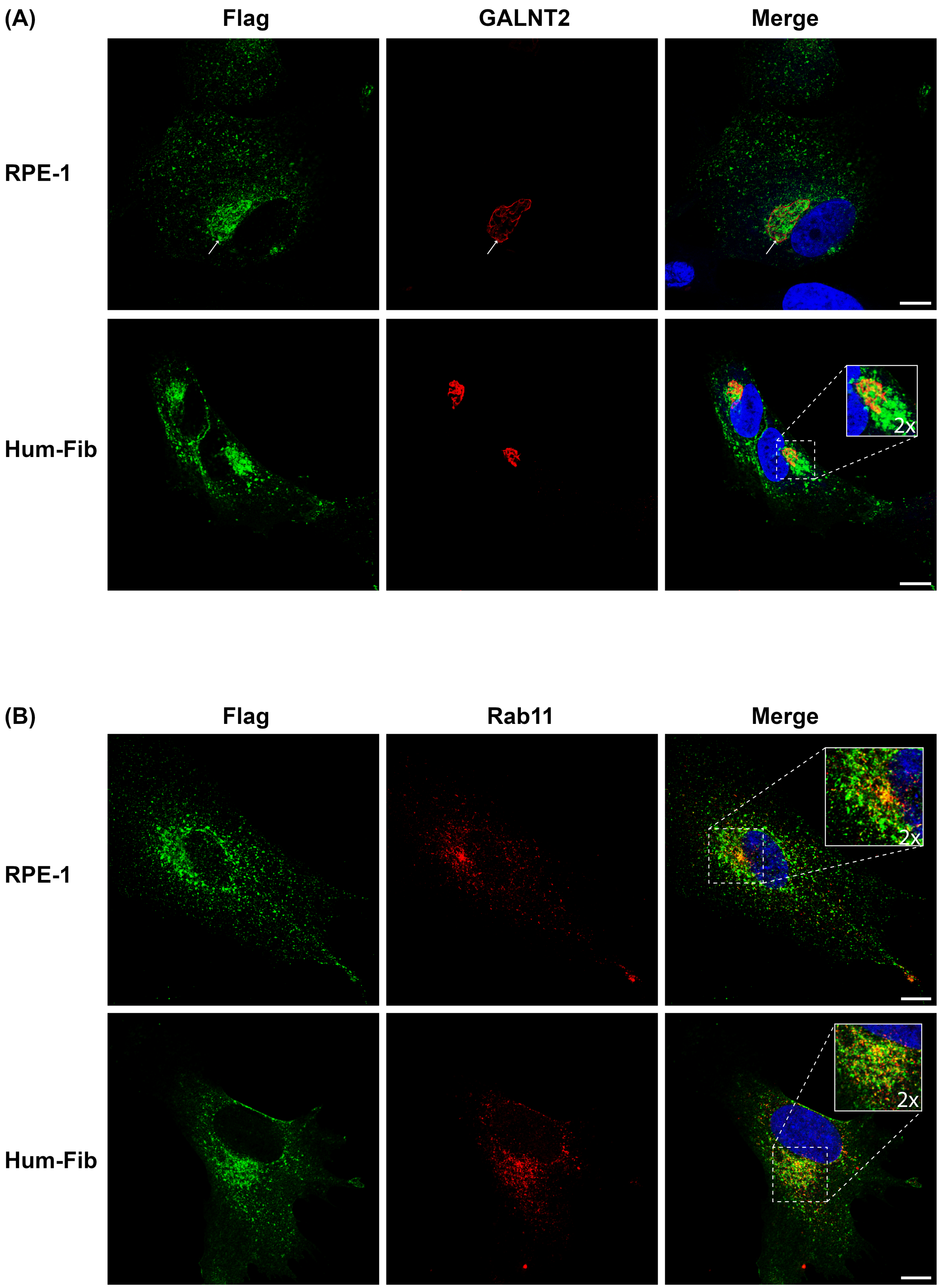

Immunofluorescence analysis of transduced RPE-1 and Human fibroblasts (Hum-Fib) cells overexpressing Myc-Flag-tagged ATRAID Iso C (complementing Figure 4). (A) Co-staining with anti-Flag and anti-GALNT2 antibodies. (B) Co-staining with anti-Flag and anti-Rab11 antibodies. Arrows and boxes indicate colocalization. Scale bar: 10 µm.

Supplementary figure S8

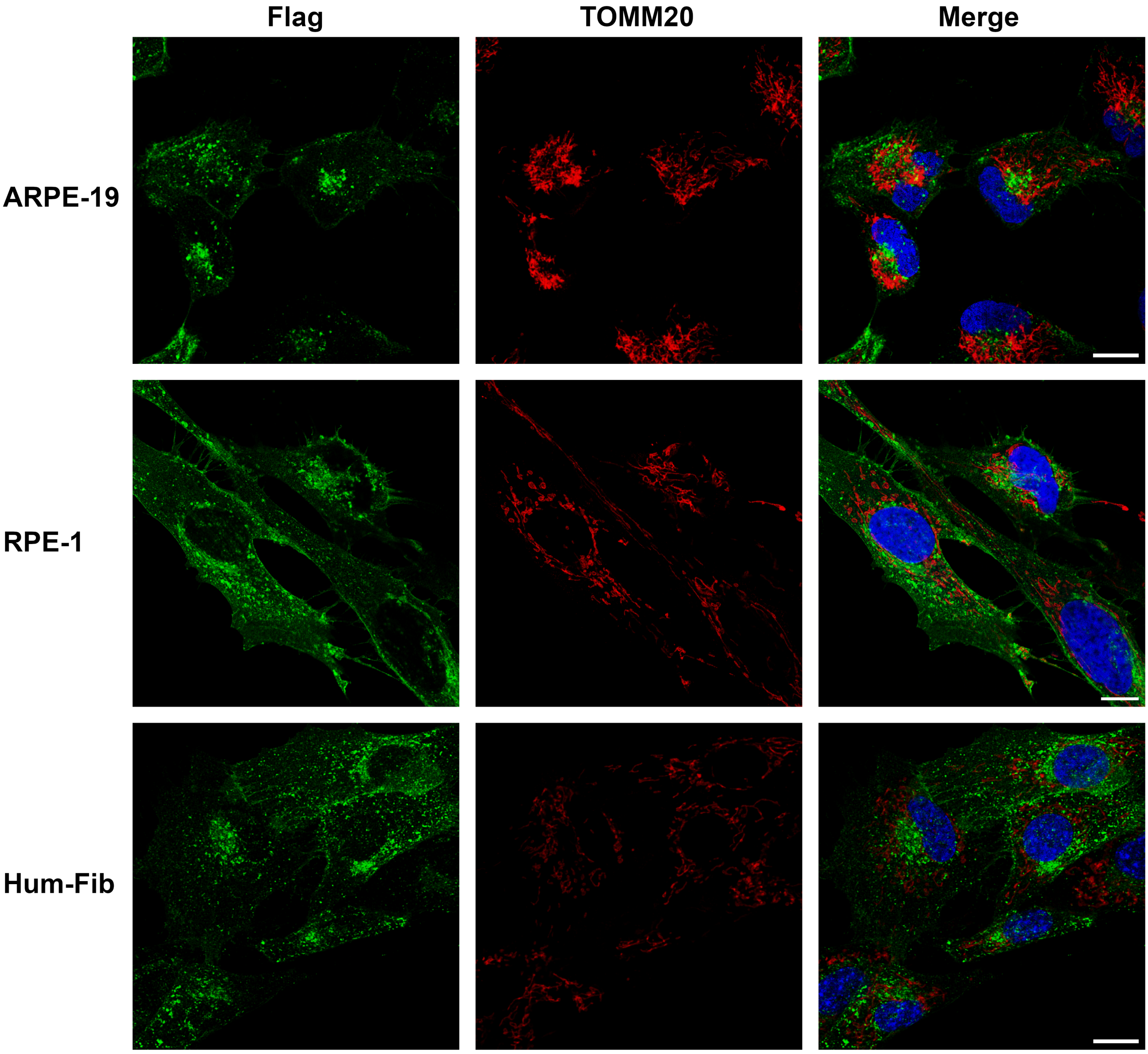

Immunofluorescence analysis of transduced ARPE-19, RPE-1 and Hum-Fib cells overexpressing Myc-Flag-tagged ATRAID Iso C. Co-staining with anti-Flag and anti-TOMM20 antibodies. Merged images show no colocalization between TOMM20 and ATRAID Iso C. Blue is DAPI staining. Scale bar: 20 μm. Scale bar of ARPE-19 and Hum-Fib is 10 μm, and the scale bar of RPE-1 is 40 μm.

Supplementary Table S1

Name	Sequence, 5->3'	Target	Purpose
IsoA F	TCGACTGGATCCGGTACCGAG	Origene RC203440	Subcloning to pQCXIP
IsoA R	GCAAGAATTCGCCGTTTAAACCTTATCGTC		
IsoC F	TAACGGGATCCGATCGCCATGGCGCCTCACGACCCG	Origene RC229723	
IsoC R	GACGAATTCTTAAACCTTATCGTCGTCATC		

DNA sequence of PCR primers for subcloning ATRAID Iso A and Iso C from Origene expression vectors to the murine retroviral vector pQCXIP
